# Evaluation of immunogenicity and protective efficacy of recombinant outer membrane proteins of *Haemophilus parasuis* serovar 5 in a murine model

**DOI:** 10.1371/journal.pone.0176537

**Published:** 2017-04-27

**Authors:** Miao Li, Ru-Jian Cai, Shuai Song, Zhi-Yong Jiang, Yan Li, Hong-Chao Gou, Pin-Pin Chu, Chun-Ling Li, Hua-Ji Qiu

**Affiliations:** 1 Institute of Animal Health, Guangdong Academy of Agricultural Sciences, Guangzhou, China; 2 Guangdong Open Laboratory of Veterinary Public Health, Guangzhou, China; 3 Guangdong Provincial Key Laboratory of Livestock Disease Prevention, Guangzhou, China; 4 State Key Laboratory of Veterinary Biotechnology, Harbin Veterinary Research Institute, Chinese Academy of Agricultural Sciences, Harbin, China; Universidad Nacional de la Plata, ARGENTINA

## Abstract

Glässer’s disease is an economically important infectious disease of pigs caused by *Haemophilus parasuis*. Few vaccines are currently available that could provide effective cross-protection against various serovars of *H*. *parasuis*. In this study, five OMPs (OppA, TolC, HxuC, LppC, and HAPS_0926) identified by bioinformatic approaches, were cloned and expressed as recombinant proteins. Antigenicity of the purified proteins was verified through Western blotting, and primary screening for protective potential was evaluated *in vivo*. Recombinant TolC (rTolC), rLppC, and rHAPS_0926 proteins showing marked protection of mice against *H*. *parasuis* infection, and were further evaluated individually or in combination. Mice treated with these three OMPs produced humoral and host cell-mediated responses, with a significant rise in antigen-specific IgG titer and lymphoproliferative response in contrast with the mock-immunized group. Significant increases were noted in CD4^+^, CD8^+^ T cells, and three cytokines (IL-2, IL-4, and IFN-γ) in vaccinated animals. The antisera against candidate antigens could efficiently impede bacterial survival in whole blood bactericidal assay against *H*. *parasuis* infection. The multi-protein vaccine induced more pronounced immune responses and offered better protection than individual vaccines. Our findings indicate that these three OMPs are promising antigens for the development of multi-component subunit vaccines against Glässer's disease.

## Introduction

*Haemophilus parasuis* is an early colonizer of the upper respiratory tract of pigs, and the etiological agent of Glässer's disease, which is characterized by polyserositis, polyarthritis, meningitis, and arthritis [1–2]. Glässer's disease has been reported sporadically and is usually associated with precipitating stress factors. With the effects of immunosuppressive viruses and increasingly intensive swine production, there is an apparent increase in the prevalence of the disease [3]. Nowadays, *H*. *parasuis* is a widespread epidemic pathogenic bacteria and leads to huge economic losses to the world swine industry, while prevention and control of Glässer's disease remain a big challenge [4].

Fifteen distinct serovars of *H*. *parasuis* have been described, while approximately 26% of the isolates were reported as non-typeable using traditional serotyping [5–6], and this percentage was lower when detected by molecular serotyping methods [7–8]. Commercial vaccines mainly comprise inactivated whole-cell vaccines and could not confer cross-protection against different serovars [9]. Currently, the development of subunit vaccines has attracted more attention, and they mainly concentrate upon outer membrane proteins (OMPs) as vaccine candidate antigens [10]. OMPs are unique to Gram-negative bacteria and have been shown to be potential candidates for vaccine development against infections in recent years [11]. Several OMPs of *H*. *parasuis*, such as PalA, D15, OmpP2, HPS-06257, OapA, HPS-0675, GAPDH, native outer membrane proteins with affinity to porcine transferrin (NPAPT) and many more have been confirmed to exhibit a strong potential as vaccine candidates [12–16]. However, a combination of protective antigens may be able to provide effective protection against multiple *H*. *parasuis* serovars.

In our previous study, six secreted proteins and seven OMPs were predicted using bioinformatic analysis and were evaluated as potential vaccine candidates of *H*. *parasuis* serovar 5 [17–18]. In the present study, we adopted the same approach to identify protective antigens. Five OMPs, including OppA (oligopeptide permease ABC transporter membrane protein), TolC (RND efflux system outer membrane lipoprotein), LppC (lipoprotein C), HAPS_0926 (DNA uptake lipoprotein), and HxuC (haem-haemopexin utilization protein C/outer membrane receptor protein) were cloned, expressed, and purified, and initially screening for protective potential was performed in a murine model. Then rTolC, rLppC, and rHAPS_0926, showing a good protective potential, were administered individually or in combination to evaluate the protective immunity against *H*. *parasuis*.

## Materials and methods

### Ethics statement

All animal procedures were approved by the Ethics Committee of Institute of Animal Health, Guangdong Academy of Agricultural Sciences according to Guangdong Province Laboratory Animal Management Regulations—2010. The license number was SYXK(Yue) 2011–0116. All efforts were made to minimize suffering. Humane endpoints used during the animal survival study were: rapid weight loss of >20% of body weight, poor physical appearance (reduced mobility, rough coat and depression), rapid breathing, swollen eyes, and joint tumefaction. Following infection, the healthy status of animals was evaluated every 8 h and there were not unexpected deaths. Animals that reached humane endpoints were euthanized through complete exsanguination via cardiac puncture under general anesthesia with inhaled 2% isoflurane.

### Bacterial strains and growth conditions

*H*. *parasuis* was maintained in tryptic soy broth (TSB) (Difco, Detroit, MI, USA) with 10% inactivated newborn calf serum and 0.01% nicotinamide adenine dinucleotide (NAD) (Sigma, St. Louis, MO, USA) or plated on tryptic soy agar (TSA) (Difco, USA) plus 10% serum, and 0.01% NAD at 37°C. *Escherichia coli* strains were cultured in Luria-Bertani (LB) medium. If necessary, 100 μg/mL ampicillin or 50 μg/mL kanamycin was complemented. *E*. *coli* DH5α (Invitrogen, Carlsbad, CA, USA) and BL21(DE3) (Invitrogen, USA) were used for the cloning of plasmids and expression of recombinant proteins. The *H*. *parasuis* serovar 5 H46 was isolated from a pig farm in Guangdong Province, China [18]. For challenge test, H46 was cultured on TSA agar for 16 h at 37°C. Then a single clone was randomly picked, inoculated into 5 mL TSB (plus 10% serum and 0.01% NAD) and shaken at 37°C overnight. The overnight culture was reinoculated into 500 mL of TSB medium to bacteria counting.

### Screening, cloning, expression, and purification of the recombinant proteins

To identify the OMPs of *H*. *parasuis* serovar 5 as candidate vaccines, we used a strategy combining bioinformatic analysis with an experimental approach as described previously [17]. The gene sequences of five selected antigens (OppA, TolC, LppC, HAPS_0926, and HxuC) were collated from *H*. *parasuis* SH0165 complete genome sequence [19].

Total genomic DNA was prepared from an *H*. *parasuis* serovar 5 H46 strain. Briefly, H46 was cultured in TSB overnight and 5 mL of culture was collected and lysed with the Bacterial DNA extraction kit (Sangon, Shanghai, China). The DNA region encoding each protein without the putative secreted signal was amplified with the primer pairs (Table 1) from H46 genomic DNA. The amplified PCR products digested with restriction enzymes (*BamH* I/*Xho* I) were used to transform into the pET-30a(+) vector (Novagen, Billerica, MA, USA), and then were transformed into *E*. *coli* DH5α. The plasmid constructs were verified by restriction digestion, PCR, and sequencing. The constructed expression vectors were transformed to *E*. *coli* BL21(DE3) for expression with a C-terminal 6 × His-tag. Recombinant proteins were induced upon treatment with 0.6 mM isopropyl-β-d-thiogalactopyranoside (IPTG) for 4 h. These expressed OMPs were purified by Ni^2+^-NTA affinity chromatography (Qiagen, Dusseldorf, Germany) in accordance with the instructions. Protein quantifications were detected with the bicinchoninic acid (BCA) protein assay kit (Tiangen, Beijing, China) and stored at –80°C.

**Table 1:** Experimental design for grouping and antigen dose of immunization.

Group	Vaccine	Dose
1	rTolC	60 μg/200 μL
2	rLppC	60 μg/200 μL
3	rHAPS_0926	60 μg/200 μL
4	rTolC + rLppC	30μg each/200 μL
5	rTolC + rHAPS_0926	30μg each/200 μL
6	rLppC + rHAPS_0926	30μg each/200 μL
7	rTolC + rLppC+ rHAPS_0926	20 μg each/200 μL
8	PBS	200 μL

### SDS-PAGE and Western blotting analysis

Five purified recombinant OMPs were subjected to sodium dodecyl sulfate polyacrylamide gel electrophoresis (SDS-PAGE), and the transfer of proteins to a polyvinylidene fluoride (PVDF) membrane. Western blotting analysis was carried out using our previous study [17–18]. Convalescent swine sera (1:500 diluted with 5% skim milk in PBST) were added and incubated for 1 h at room temperature (RT) as primary antibody, and goat anti-porcine IgG (H + L)-HRP antibody (1:5,000) (Sigma, USA) was added and incubated at RT for 1 h as secondary antibody. After washing three times with PBS, the specific antigen-bound antibody was visualized with ECL (Biovision, Milpitas, CA, USA) following the manufacturer’s instructions.

### Immunization-challenge test of the selected five OMPs in mice

All animals used in this study were maintained under optimal conditions of temperature, hygiene, humidity, and light with a 12-h dark/light cycle. Sixty female BALB/c mice (seven-week-old) were randomly divided into six groups of 10 mice each. Five groups were vaccinated subcutaneously with 50 μg each of recombinant OMP emulsified in 200 μL of complete Freund’s adjuvant (CFA) (Sigma, USA). The mice were boosted at 21 days post-immunization (dpi) using the identical dosage of antigens, but with incomplete Freund’s adjuvant (IFA) (Sigma, USA). The sixth group, serving as a negative control (NC), was immunized with phosphate-buffered saline (PBS) emulsified in the corresponding Freund’s adjuvant. At 42 days following the first immunization, all of the mice were intraperitoneally infected with 2 × 10^9^ colony forming unit (CFU) log-phase *H*. *parasuis* H46 strain. After the challenge, clinical signs were monitored for two weeks, and mice judged to be in a moribund state were euthanized.

### Evaluation of the efficacy of the three screened OMPs in mice

One hundred and twenty mice were randomly assigned to eight groups of 15 mice each. Three screened OMPs rTolC, rLppC, and rHAPS_0926 were placed in eight groups (Table 2). Groups 1 to 3 were immunized with 60 μg of each recombinant protein emulsified in 200 μL of CFA via subcutaneous injection. Groups 4 to 6 received a mixture of two kinds of the recombinant OMPs (containing 30 μg of each antigen) with an equal volume of CFA. Group 7 was immunized with mixed rTolC, rLppC, and rHAPS_0926 (20 μg each). Mice in Group 8 were immunized with 200 μL of PBS emulsified in CFA serving as a NC. A booster immunization was given at 21 dpi using the same antigen and IFA.

**Table 2:** Primers for amplifying the genes encoding the five outer membrane proteins.

Genes	Primer sequences^a^
*OppA*	GC*GGATCC*TTATTAGCCAGTGCGATT
	GCC*CTCGAG*TTACTGCTTAATGATATA
*TolC*	GC*GGATCC*TTACTTTCTGCACTGGTA
	CC*CTCGAG*CTATTTGCGATATTTCCC
*LppC*	GC*GGATCC*ACCGCCACGATATTGTAG
	GG*CTCGAG*GTTTGCATCAACAATAGA
*HxuC*	GC*GGATCC*ATTTAATATTGCGCCCAG
	GCC*CTCGAG*ATGAGACTATCAAAAATT
*HAPS_0926*	GCC*GGATCC*ATATTTGTAAAGGTTTTA
	GG*CTCGAG*AAATTGCTCGCCATATTT

^a^ Introduced restriction sites were highlighted in italics and underlined.

At 42 days following the primary immunization, the immunized mice (ten mice in each group) were intraperitoneally inoculated with 5.0 × 10^9^ CFU log-phase *H*. *parasuis* H46 strain. The animals were intensively monitored daily after the challenge for the presence and severity of clinical symptoms of illness or mortality. At 14 days post-challenge (dpc), all surviving mice were euthanized, and different tissues (heart, liver, spleen, lung, and kidney) were collected and subjected to pathological and immunohistochemistry (IHC) examinations and PCR.

### Indirect ELISA

An indirect enzyme-linked immunosorbent assay (ELISA) was made in accordance with the method described in previous study [13]. The sera collected from pre-immune mice by tail vein bleeding were used as controls. Briefly, ELISA plates were coated with 0.2 μg/well of purified recombinant protein antigen diluted in coating buffer (0.02 M carbonate-bicarbonate buffer, pH 9.6), incubated overnight at 4°C. Following washing, 1% (w/v) bovine serum albumin in PBST (PBS containing 0.05% Tween-20, pH 7.4) was added to wells for blocking. Then the plates were incubated with serially diluted serum samples (initially in 1:100) at 37°C for 1 h. Horseradish peroxidase-conjugated goat anti-mouse IgG (Sigma, USA), at a dilution of 1:5,000, was subsequently added to incubate at 37°C for 1 h. The tetramethylbenzidine was added for 10 min in the dark and then 2 M H_2_SO_4_ was added to stop the reaction. The absorbance was read at optical density of 450 nm (OD_450 nm_) with an ELISA plate reader (Bio-Rad, Hercules, CA, USA). Antibody titer was calculated as the reciprocal of the maximum serum dilution that gave an OD yielding the cutoff value of ELISA (OD_450 nm_ = 0.35, which is the mean values of negative sera plus three-fold standard errors).

### Flow cytometry

Fourteen days after the booster immunization, mice were killed and aseptically collected spleens were washed in PBS (pH 7.4). The splenocytes were harvested from the immunized mice as described previously [17,20]. Lymphocyte subtype analysis was performed as described previously [21]. Briefly, splenocytes were labeled with fluorescein isothiocyanate (FITC)-anti-mouse CD4^+^, allophycocyanin-cyanine 7 (APC-Cy7)-anti-mouse CD3^+^, and phycoerythrin (PE)-anti-mouse CD8^+^. FITC-, APC-Cy7-, or PE-conjugated antibodies were used as isotype controls (eBioscience, San Diego, CA, USA). Then the percentage of CD3^+^, CD4^+^, and CD8^+^ T cells was quantified.

### Lymphocyte proliferation assay

Spleen lymphocytes resuspended in RPMI 1640 complete medium (Gibco, USA) supplemented with 10% inactivated fetal bovine serum were adjusted to 10^6^cells/mL and 100 μL of the suspension was added per well in 96-well plates, and incubated with 5 μg/well recombinant proteins in 5% CO_2_ for 72 h at 37°C. Splenocytes stimulated with 5 μg/well of concanavalin A (ConA) (Sigma, USA) served as a positive control, while negative control cells received medium only. Lymphoproliferation assay was carried out with a MTS cell proliferation detection kit (Promega, Madison, WI, USA). The lymphocytes were incubated with MTS reagent for 4 h. The proliferation of cells was measured at OD_490 nm_ using a plate reader (Bio-Rad, USA).

### Cytokine assay

Cytokine assay was performed as described previously [17]. Supernatants obtained in Section of lymphocyte proliferation assay were harvested and stored at ÿ80°C. Interleukin 2 (IL-2), IL-4, and interferon gamma (IFN-γ) levels were measured using cytokine detection kits following the manufacturer’s instructions (Research & Diagnostics Systems, Minneapolis, MN, USA).

### *In vitro* whole blood bactericidal assay

The whole blood bactericidal assay was carried out as described previously [18]. The H46 cultures were washed three times and diluted in sterilized PBS to 10^8^ CFU. Subsequently, a mixture of 10 μL of H46 suspension and 190 μL of each serum was incubated for 30 min at 37°C. Then 100 μL of nonimmune heparinized blood was added and incubated for 1 h at 37°C with shaking. The samples were plated on TSA plates and colonies were determined after 24 h. The resulting expression was performed as described previously [18].

### Bacterium re-isolation from the immunized mice following challenge

At 1, 2, and 7 dpc, the presence of *H*. *parasuis* in the liver, spleen, and lung was detected. The tissue sections were prepared as described previously [18]. Then serial 10-fold dilution samples were determined by plating on TSA plates and incubated for 16 h at 37°C.

### Statistical analysis

The immunized groups were compared with the negative group. Descriptive statistics (mean, standard error), normality (Shapiro-Wilk test), and homoscedasticity (Bartlett’ s test) were determined. Data were analyzed by ANOVA test using the software Statistics Package for Social Science19.0 (SPSS 19.0).

## Results

### Expression and purification of five recombinant OMPs

**Fig 1:**
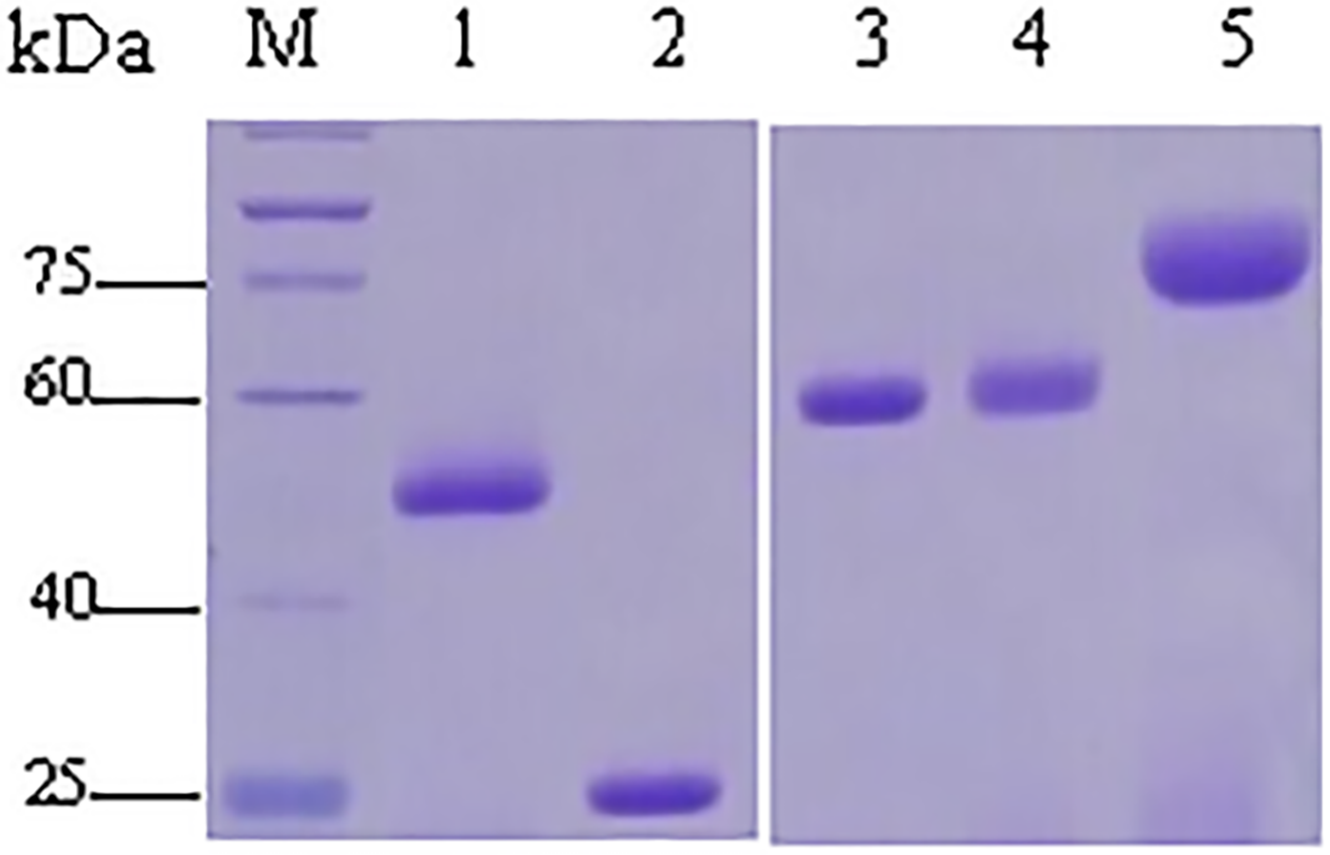
SDS-PAGE analysis of the *E*. *coli*-expressed five recombinant proteins purified by Ni^2+^-NTA affinity chromatography. Lane M: protein marker; Lanes 1–6: TolC (approx. 51 kDa), HAPS_0926 (approx. 25 kDa), OppA (approx. 60 kDa), LppC (approx. 62 kDa), and HxuC (approx.78 kDa).

The DNA fragments encoding the protein of OppA, TolC, LppC, HAPS_0926, and HxuC were successfully cloned into pET-30a(+), confirmed by sequencing (data not shown) and the digestion of *Bam*H I/*Xho* I (data not shown). The recombinant plasmids harboring the foreign genes were used to transform into *E*. *coli* BL21(DE3). The SDS-PAGE results indicated that all of the recombinant proteins were expressed in *E*. *coli* as His-tagged fusion proteins with an expected size (Fig 1).

### Antigenicity of five recombinant OMPs

**Fig 2:**
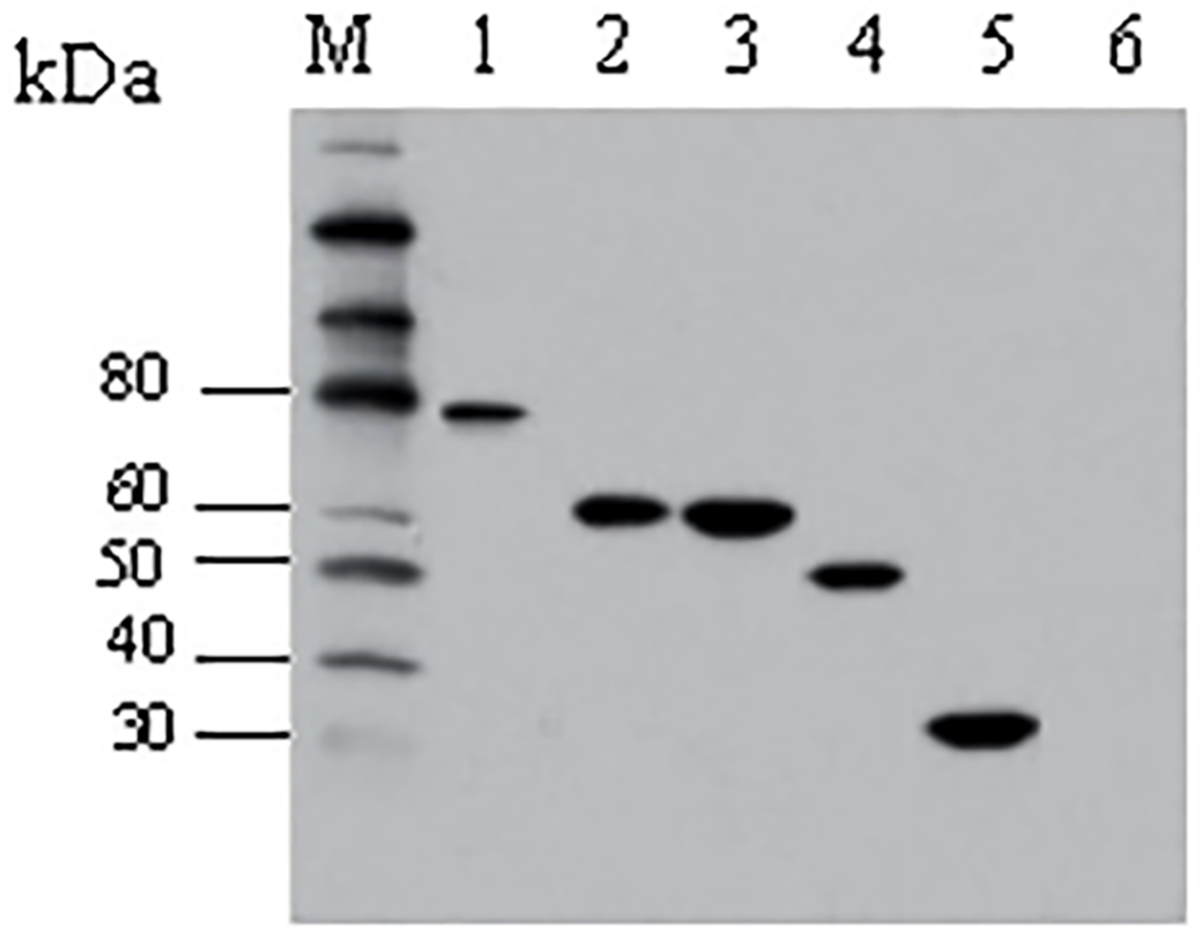
Western blotting analysis of the purified five recombinant proteins. Lanes 1–6: purified HxuC (approx.78 kDa), LppC (approx. 62 kDa), OppA (approx. 60 kDa), TolC (approx. 51 kDa), and HAPS_0926 (approx. 25 kDa); Lane 7: sonicated whole cells of *E*. *coli* serving as a NC; Lane M: protein molecular weight marker.

The expression of the five recombinant OMPs was further confirmed by Western blotting using the porcine convalescent sera to *H*. *parasuis* [18] (Fig 2). The results showed that the *H*. *parasuis* convalescent sera reacted with five recombinant proteins, indicating that the obtained recombinant proteins can be used for further evaluation of immunoprotection.

### Protective potential of the five antigens in mice

The protective potential of the five OMPs against a lethal challenge with H46 was evaluated in mice. The results indicated that rTolC, rLppC, and rHAPS_0926 provided 80%, 70%, and 60% protection, respectively. rOppA and rHxuC did not afford significant protection (survival of ≤ 50%). Therefore, in the following experiment, the immune responses and efficacy of rTolC, rLppC, and rHAPS_0926 were further evaluated separately and in combination.

### Antibody responses in the vaccinated mice

**Fig 3:**
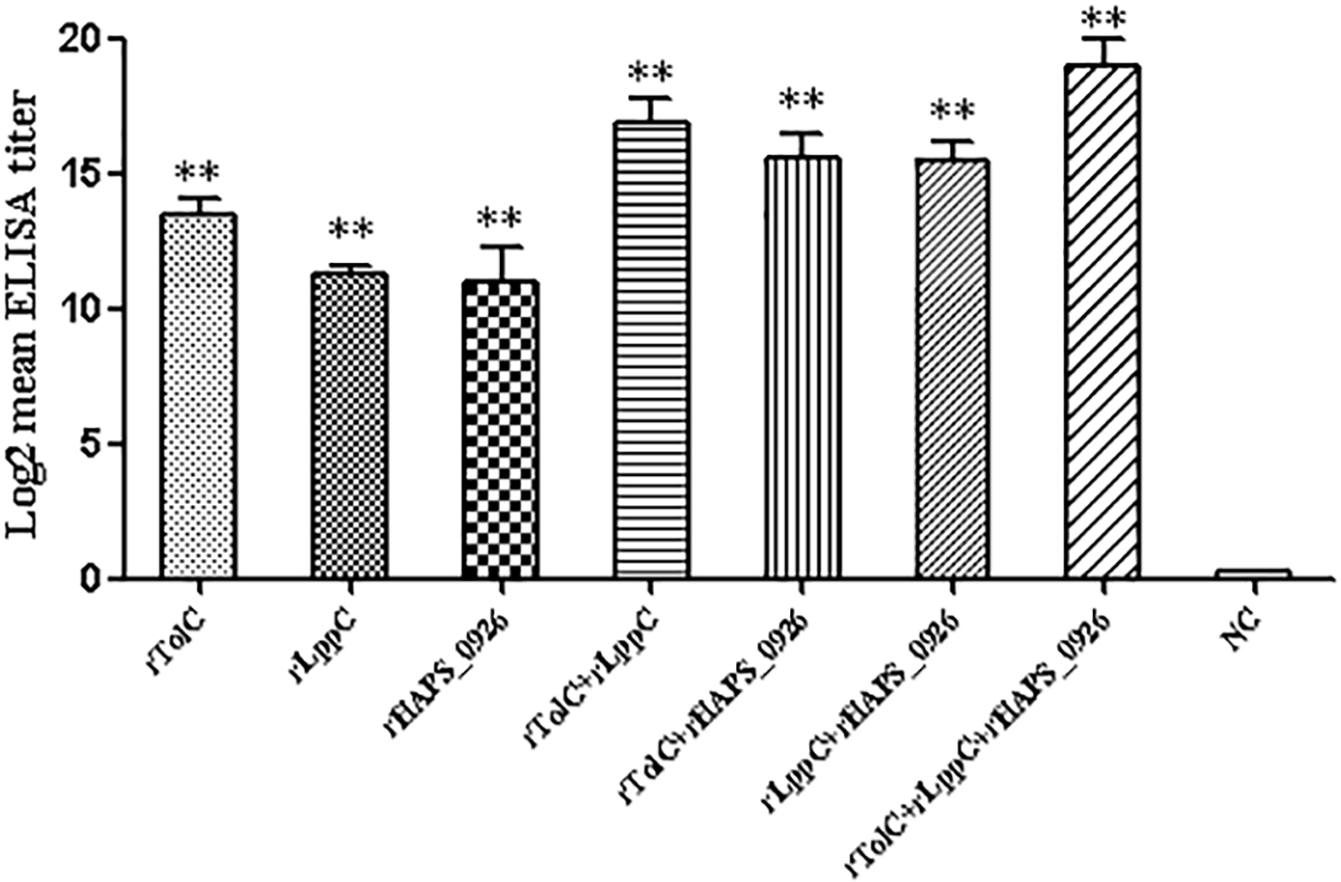
Serum IgG antibody titers against the recombinant proteins in mice. Serum samples were collected two weeks after the booster immunization and tested for the antibody titers by indirect ELISA. Standard deviations were shown as error bars. **, *p* < 0.01; ***, *p* < 0.001.

To determine whether the mice immunized with the recombinant proteins can induce specific humoral immune responses, an indirect ELISA was carried out. Serum samples from the immunized mice were collected two weeks after the booster immunization. The individual or mixed recombinant proteins were used as coating antigens. As shown in Fig 3, specific IgG antibody titers against all of the recombinant protein-immunized groups were significantly higher than that of the negative group (*p* < 0.01). The mice immunized with two antigens in combination displayed slightly higher levels of IgG titers than that of the mice immunized with single antigens. Compared to the other groups, the rTolC + rLppC + rHAPS_0926 (triple-rOMP)-immunized group developed the highest antigen-specific response.

### Cell-mediated immune responses in the immunized mice

**Fig 4:**
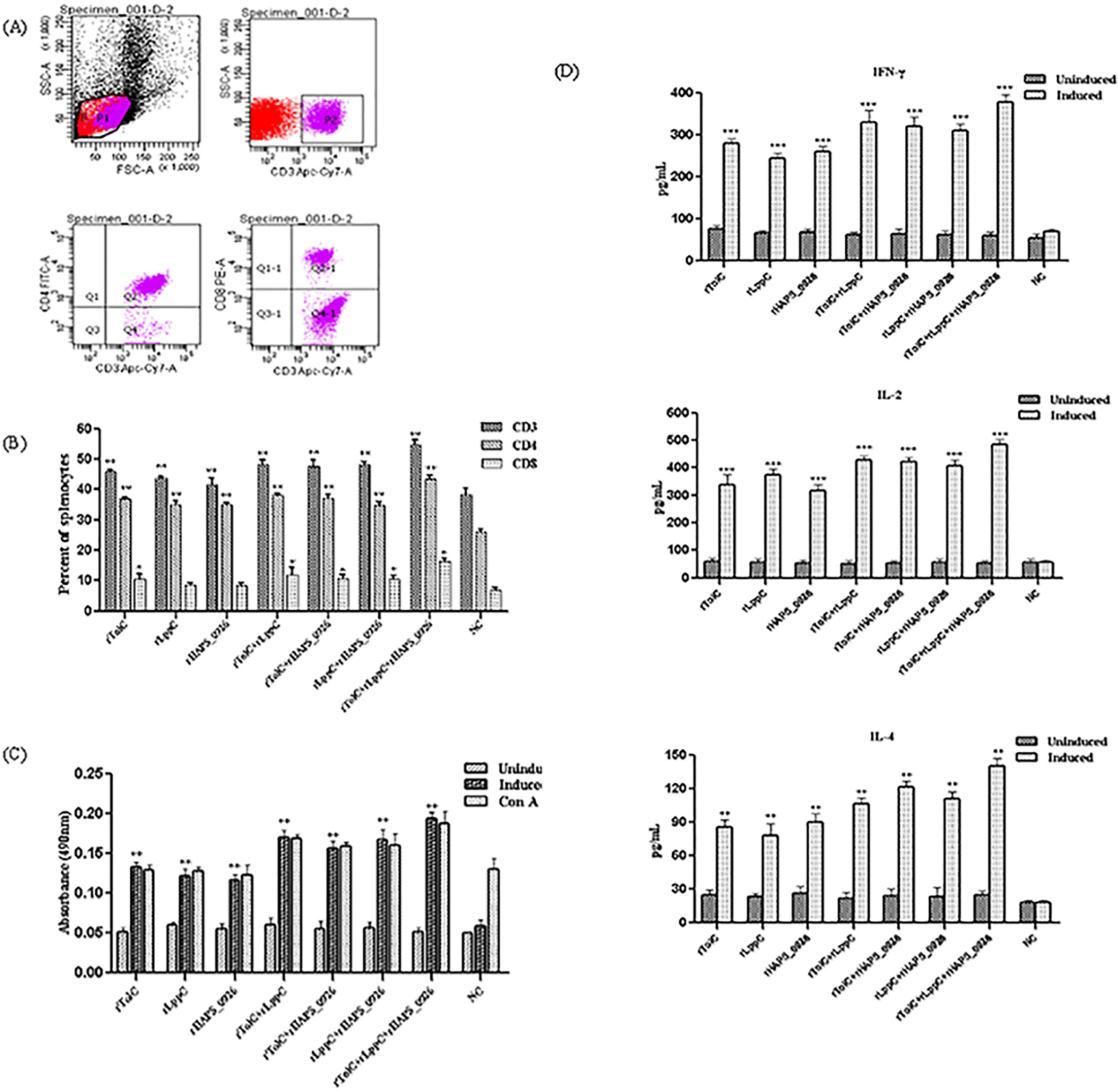
(A) Dot plot analysis of CD4^+^ and CD8^+^ T cell proliferation. (B) Expression of T lymphocyte subsets in the spleens collected from the mice immunized with recombinant proteins or PBS (NC). Splenocytes were stained with FITC-labeled anti-mouse CD4^+^ and PE-labeled anti-mouse CD8^+^ antibodies. Significant increase in CD4^+^ (*p* < 0.01) and CD8^+^ (*p* < 0.05) subsets was observed for the vaccinated group compared to the NC group. (C) Lymphocyte proliferation assay. Splenocytes from the mice immunized with the recombinant proteins or PBS (NC) were stimulated *in vitro* with the corresponding recombinant proteins for 72 h and the lymphoproliferative responses were measured by MTS assay. Stimulation with ConA serving as a positive control. (D) Expression of IFN-γ, IL-2, and IL-4 in the spleens isolated from the mice immunized with the recombinant proteins or PBS (NC). Standard deviations were shown as error bars. ***, *p* < 0.001; **, *p* < 0.01; *, *p* < 0.05.

To detect the cell-mediated immune responses, the immunized mice were sacrificed. The splenocytes isolated at two weeks following the booster immunization were analyzed using flow cytometry (Fig 4A). Compared to the NC, the proportion of proliferated CD4^+^ (*p* < 0.01) and CD8^+^ (*p* < 0.05) T cells was significantly higher for the recombinant protein-immunized groups, and the percentage of proliferated CD4^+^ T cells was much higher than that of CD8^+^ T cells. In contrast with the mice immunized with individual antigens or two antigens combination, the mice immunized with triple-rOMP displayed a very significant increase in the percentage of CD4^+^ and CD8^+^ T cells (*p* < 0.01) (Fig 4B).

As shown in Fig 4C, the seven groups of vaccinated animals showed significant antigen-specific proliferative cell immune responses (*p* < 0.01). Similarly, a strong proliferative response was determined to ConA as a positive control. However, no antigen-specific lymphoproliferation was found in the NC group.

The cytokine response of splenic lymphocytes was detected by ELISA. Compared to the NC, splenocytes from the recombinant protein-immunized mice induced a significant cytotoxic response of IFN-γ (*p* < 0.001), IL-2 (*p* < 0.001), and IL-4 (*p* < 0.01) (Fig 4D). Higher levels of IL-2 and IFN-γ were secreted than that of IL-4. These results suggested that the immunization of mice with individual antigens and multi-proteins could induce the Th1 type response.

### Bactericidal activities of the antisera from the immunized mice

**Fig 5:**
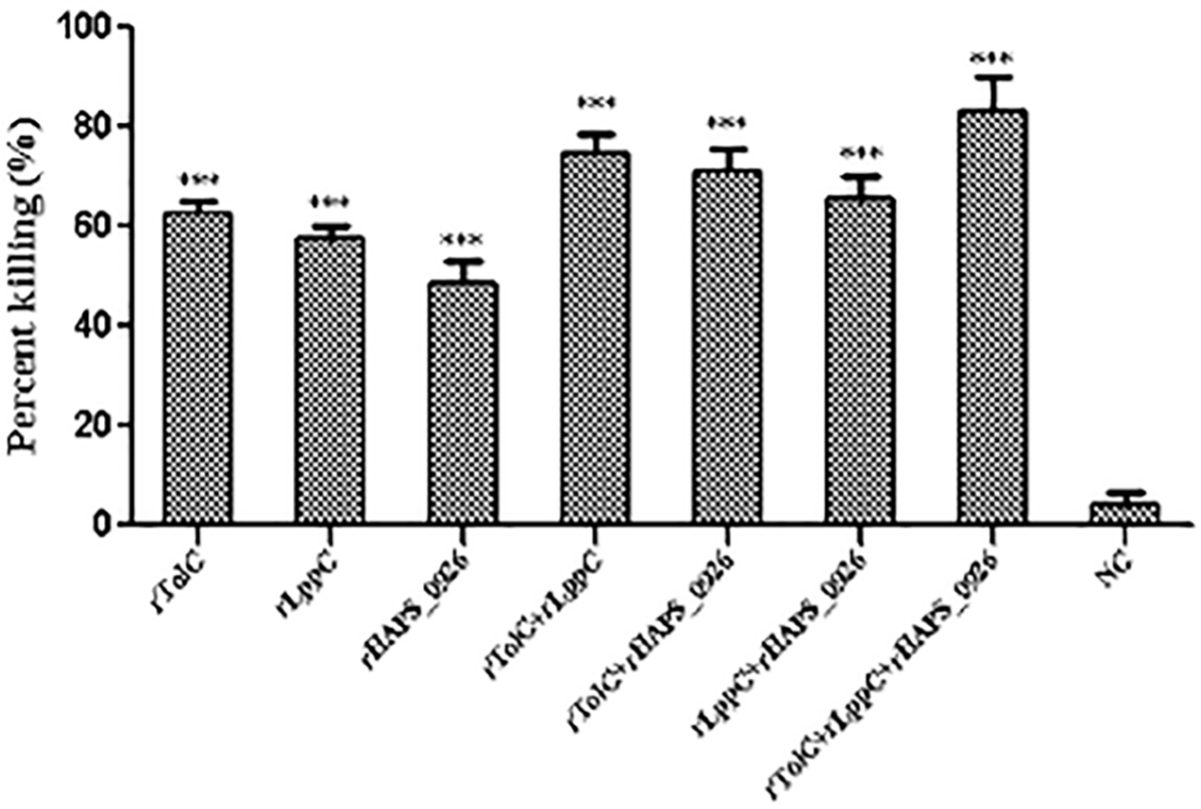
Bactericidal activities of the whole blood from the mice immunized with the recombinant proteins or PBS (NC). The results were expressed in pg/mL. Error bars represent the standard errors of the means from three replicates. ***, *p* < 0.001.

Antisera against the recombinant proteins significantly inhibited (*p* < 0.001) the growth of *H*. *parasuis* compared to the negative NC (Fig 5). Compared to the individual recombinant proteins, the bactericidal activities of antiserum of triple-rOMP-immunized mice were much higher. The results suggest that the immune responses induced by the recombinant proteins are able to provide partial protection against *H*. *parasuis* infection.

### Protection of the immunized mice from lethal *H*. *parasuis* challenge

**Fig 6:**
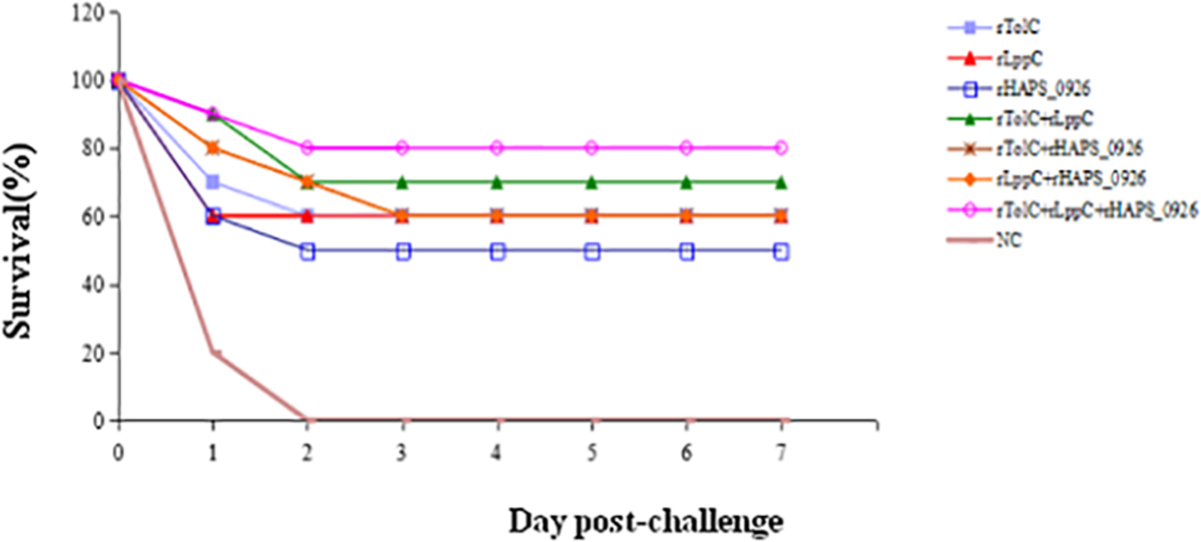
The immunized mice’ survival following challenge with *H*. *parasuis* serovar 5.

To evaluate the protective effectiveness of the three vaccine candidates, groups 1–8 were each challenged with 5.0 × 10^9^ CFU H46. The mortality and clinical signs of the mice were recorded daily for 14 dpc. As shown in Fig 6, the mice of the NC group died within 2 dpc and showed severe pathological changes, including pulmonary consolidation with massive proliferation of fibroblasts, pleura edematous, fibrin in the abdomen, and fibrin in the thorax/hydrothorax. At 14 dpc, all surviving mice were euthanized and subjected to pathological examination. None of the mice immunized with the recombinant proteins showed pathological changes (data not shown). Examination of the heart, liver, spleen, lung, and kidney by PCR[22]showed that no *H*. *parasuis* was detectable in the tissues of any animal of the vaccination groups (data not shown).

The post-challenge survival data showed that 80% of the animals survived in the triple-rOMP-immunized group. In contrast with the individual antigens, immunization with the combined three proteins provided better protection against the *H*. *parasuis* serovar 5 challenge.

### Bacterial loads in various tissues of immunized mice following challenge

**Fig 7:**
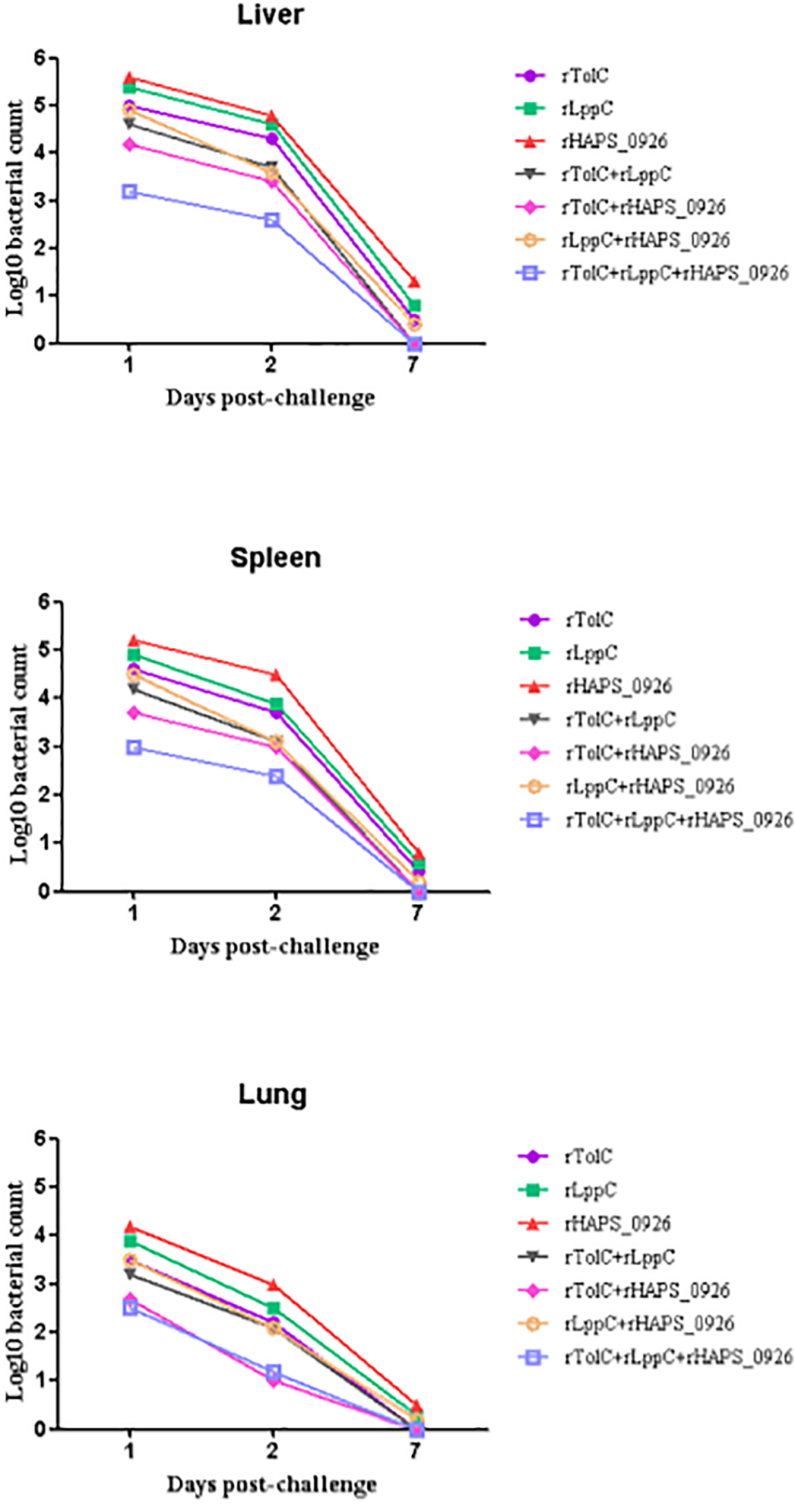
Bacterial counts in different tissues from the vaccinated animals at 1, 2, and 7 dpc following challenge with *H*. *parasuis* serovar 5. The bacterial loads in the tissues of the animals were expressed as log_10_CFU/50 μg of tissues.

As shown in Fig 7, from 1 to 7 dpc, the bacterial loads in the liver and spleen were higher than those in the lung. Analyzing the viable counts in tissues, we observed a significant reduction in the recombinant protein-immunized groups at 7 dpc, and in Groups 4, 5, and 7, there were no bacteria in the spleen, liver, or lung. Compared with others, the animals vaccinated with triple-rOMP showed lowest counts in tested tissues. All of the isolated bacteria were confirmed to be *H*. *parasuis* serovar 5 by PCR (data not shown) [22].

## Discussion

*H*. *parasuis* is an important respiratory tract pathogen causing severe infections in pigs. It is difficult to control the systemic infection of this organism because of the limitation of knowledge regarding its pathogenesis and immunogenicity [23–24]. Conventional vaccines for *H*. *parasuis* are inefficient for inducing cross-protective immunity [25]. OMPs of *H*. *parasuis* have been confirmed to have the potential for developing effective subunit vaccines [12–16,18]. The advances in whole-genome sequencing and bioinformatics techniques made it possible to search protective antigens for bacterial pathogens [13].

Animal models have widely been used to screen bacterial proteins as subunit candidate vaccines. For instance, mice are good models to evaluate the immunological parameters of *H*. *parasuis* [12–13,17]. In the present study, screening for the vaccine candidates was primarily performed in *in vivo* protection assay, and immunization with rTolC, rLppC, and rHAPS_0926 markedly protected mice from *H*. *parasuis* infection. The abilities of these three antigens to induce humoral and cell-mediated immunity and protection were further evaluated separately or in cocktails.

The TolC protein is used by both the type I protein secretion pathway and multidrug efflux pumps as a prototypical outer membrane channel component [26]. TolC participates in the formation of type I secretion systems of bacterial virulence factors, for instance, adenylate cyclase toxin (*Bordetella pertussis*), alkaline protease (*Pseudomonas aeruginosa*), and α-hemolysin (*E*. *coli*) [27]. A previous study demonstrated that efflux protein TolC plays an important role in biofilm formation in *E*. *coli* [28]. It is one of the most potent antigens of *Salmonella paratyphi A* and a promising candidate target for the development of new vaccines [29]. Both of LppC and HAPS_0926 are outer membrane lipoproteins. Lipoproteins are widely distributed in Gram-negative bacteria, which are involved in diverse mechanisms of physiology/pathogenesis and are considered potential target antigens for vaccine development against many infectious diseases [30–31]. Our previous study demonstrated that the VacJ lipoprotein was a good vaccine candidate against Glässer's disease [18]. We chose these three OMPs for further analysis of immunogenicity and immunoprotection. The results revealed that the mice immunized with rTolC, rLppC, or rHAPS_0926 alone provided partial protection against *H*. *parasuis* infection.

The advantage of a multivalent subunit vaccine is that it would provide better protection than the univalent component because of its ability to generate abundant immunogens. In previous studies, researchers have used a 1:1 or 1:1:1 ratio of single proteins to evaluate the efficacy of subunit vaccine candidates [24, 32]. Hence, we chose the same ratio of single OMPs to compose the multi-protein vaccines.

In the current study, these subunit vaccines were shown to be capable of inducing a high titer of antibodies and cell-mediated immunity in mice, providing strong protective potential. Similar to the previous reports, our results showed that the combination of recombinant antigens induced higher antibody titers, confirming that the antigen cocktails are more immunogenic than the individual ones [33–35]. The induction of cell-mediated immune responses characterized by antigen-specific T cell proliferation revealed the immunogenicity of multi-proteins with a higher proliferation index. The splenocytes from the multi-proteins-immunized groups secreted higher levels of cytokines in response to corresponding antigens than those of the single-components vaccinated groups, which is consistent with the lymphocyte proliferation. It was possible to verify that the multi-protein vaccines induced specific Th1-mediated immune protection in the immunized animals, as demonstrated by the specific production of IL-2 and IFN-γ *in vitro*, along with a low expression of IL-4. Our results of immunogenicity proved that the multi-protein vaccine was better than individual proteins. After the challenge, bacterial loads in all evaluated tissues were decreased significantly in the triple-rOMP-immunized mice, which were related to bactericidal activities of the antisera.

Besides the three vaccine candidates, two other proteins, OppA and HxuC, should not be ignored. OppA is an oligopeptide permease ABC transporter membrane protein, which has been identified as a vaccine development target against several pathogenic bacteria, such as *Yersinia pestis* and *Moraxella catarrhalis* [36–37]. Recently, the researchers used recombinant OppA as the antigen of an indirect hemagglutination assay for detecting antibodies against *H*. *parasuis* [38]. The TonB-dependent haem receptor HxuC belongs to the *hxuCBA* gene cluster, which is a virulence factor of *H*. *Influenzae* [39]. The results revealed that these two proteins could react with convalescent sera, but did not induce effective protection against *H*. *parasuis* infection in the mouse model.

It is important to optimize the dose and route of administration for evaluating new antigens. Development of cross-protective vaccines is needed urgently for the control and prevention against *H*. *parasuis* infection, because single antigens are insufficient to offer complete protection. Additional studies to evaluate the triple-rOMP vaccine against other serovars of *H*. *parasuis*, such as serovars 1, 4, 12, and 13, should also be performed in future studies. Indeed, subunit vaccines should be tested for the protection efficacy conferred in practice in target animals. Piglets are quite suitable for evaluating the protective potency against the heterologous challenge of *H*. *parasuis*. In previous studies, our group had already screened four secreted proteins (RnfC, Ndk, Gcp, and HsdS) and three OMPs (Omp26, VacJ, and HAPS_0742) as vaccine candidates against Glässer's disease[18,22]. Therefore, we will further evaluate the screened bacterial proteins in cocktails in pigs, which might contribute in the development of an efficacious multi-protein vaccine against Glässer's disease.

In summary, humoral and cellular immunity and protection efficacy of rTolC, rLppC, and rHAPS_0926 identified by a selective bioinformatic approach were evaluated in this study. Our results showed that three recombinant proteins elicited high-titer antibodies, T cell-mediated immunity, and substantial protection (survival rate ≥ 50%) against *H*. *parasuis* serovar 5 infection. The combination of three antigens elicited a much stronger immune response and effective protection. Therefore, an antigen cocktail containing triple-rOMP would be a valuable candidate for developing an efficient and improved multivalent subunit vaccine against *H*. *parasuis* serovar 5 infection.

## Supporting information

S1 File The excel data file used to generate the Fig 4C and 4D in this manuscript. (XLS)

## References

[1] Cai X, Chen H, Blackall PJ, Yin Z, Wang L, Liu Z, et al Serological characterization of *Haemophilus parasuis* isolates from China. Vet Microbiol. 2005; 111:231–236. 10.1016/j.vetmic.2005.07.007 16271834

[2] Oliveira S, Pijoan C. *Haemophilus parasuis*: new trends on diagnosis, epidemiology and control. Vet Microbiol. 2004; 99:1–12. 10.1016/j.vetmic.2003.12.001 15019107

[3] Aragon V, Segalés J, Oliveira S. Glässer's disease *In*: Zimmerman JJ, Karriker LA, Ramirez A, Schwartz KJ, Stevenson GW (Eds.), Diseases of Swine, 10th Ed. Wiley-Blackwell, Iowa, USA 2012; pp.760–769.

[4] Costa-Hurtado M, Aragon V. Advances in the quest for virulence factors of *Haemophilus parasuis*. Vet J. 2013; 198:571–576. 10.1016/j.tvjl.2013.08.027 24084037

[5] Kielstein P, Rapp-Gabrielson VJ. Designation of 15 serovars of *Haemophilus parasuis* on the basis of immunodiffusion using heat-stable antigen extracts. J Clin Microbiol. 1992; 30: 862–865. 1572971 10.1128/jcm.30.4.862-865.1992 PMC265175

[6] Brockmeier SL, Loving CL, Mullins MA, Register KB, Nicholson TL, Wiseman BS, et al Virulence, transmission, and heterologous protection of four isolates of *Haemophilus parasuis*. Clin Vaccine Immunol. 2013; 20:1466–1472. 10.1128/CVI.00168-13 23885030 PMC3889593

[7] Ma L, Wang L, Chu Y, Li X, Cui Y, Chen S, et al Characterization of Chinese *Haemophilus parasuis* isolates by traditional serotyping and molecular serotyping methods. PLoS One. 2006; 11:e0168903. 10.1371/journal.pone.0168903 PMC5179118 28005999

[8] Howell KJ, Peters SE, Wang J, Hernandez-Garcia J, Weinert LA, Luan SL, et al Development of a multiplex PCR assay for rapid molecular serotyping of *Haemophilus parasuis*. J Clin Microbiol. 2015; 53:3812–3821. 10.1128/JCM.01991-15 26424843 PMC4652097

[9] Miniats OP, Smart NL, Ewert E. Vaccination of gnotobiotic primary specific pathogen-free pigs against *Haemophilus parasuis*. Can J Vet Res. 1991; 55:33–36. 1832078 PMC1263410

[10] Pan J, Li C, Ye Z. Immunoproteomic approach for screening vaccine candidates from bacterial outer membrane proteins. Methods Mol Biol. 2016; 1404:519–528. 10.1007/978-1-4939-3389-1_34 27076320

[11] Huang X, Li Y, Fu Y, Ji Y, Lian K, Wei J, et al Cross-protective efficacy of recombinant transferrin-binding protein A of *Haemophilus parasuis* in guinea pigs. Clin Vaccine Immunol. 2013; 20:912–919. 10.1128/CVI.00621-12 23616407 PMC3675969

[12] Zhou M, Guo Y, Zhao J, Hu Q, Hu Y, Zhang A, et al Identification and characterization of novel immunogenic outer membrane proteins of *Haemophilus parasuis* serovar 5. Vaccine. 2009; 27:5271–5277. 10.1016/j.vaccine.2009.06.051 19576561

[13] Fu S, Zhang M, Xu J, Ou J, Wang Y, Liu H, et al Immunogenicity and protective efficacy of recombinant *Haemophilus parasuis* SH0165 putative outer membrane proteins. Vaccine. 2013; 31:347–353. 10.1016/j.vaccine.2012.11.003 23149270

[14] Frandoloso R, Martínez S, Rodríguez-Ferri EF, García-Iglesias MJ, Pérez-Martínez C, Martínez-Fernández B, et al Development and characterization of protective *Haemophilus parasuis* subunit vaccines based on native proteins with affinity to porcine transferrin and comparison with other subunit and commercial vaccines. Clin Vaccine Immunol. 2011; 18:50–58. 10.1128/CVI.00314-10 20926701 PMC3019774

[15] Martín de la Fuente AJ, Gutiérrez Martín CB, Pérez Martínez C, García Iglesias MJ, Tejerina F, Rodríguez Ferri EF. Effect of different vaccine formulations on the development of Glässer's disease induced in pigs by experimental *Haemophilus parasuis* infection. J Comp Pathol. 2009; 140:169–176. 10.1016/j.jcpa.2008.10.007 19135210

[16] Martín de la Fuente AJ, Rodríguez-Ferri EF, Frandoloso R, Martínez S, Tejerina F, Gutiérrez-Martín CB. Systemic antibody response in colostrum-deprived pigs experimentally infected with *Haemophilus parasuis*. Res Vet Sci. 2009; 86:248–253. 10.1016/j.rvsc.2008.07.017 18783805

[17] Li M, Song S, Yang D, Li C, Li G. Identification of secreted proteins as novel antigenic vaccine candidates of *Haemophilus parasuis* serovar 5. Vaccine. 2015; 33:1695–1701. 10.1016/j.vaccine.2015.02.023 25704800

[18] Li M, Li C, Song S, Kang H, Yang D, Li G. Development and antigenic characterization of three recombinant proteins with potential for Glässer's disease prevention. Vaccine. 2016; 34:2251–2258. 10.1016/j.vaccine.2016.03.014 26993332

[19] Yue M, Yang F, Yang J, Bei W, Cai X, Chen L. Complete genome sequence of *Haemophilus parasuis* SH0165. J Bacteriol. 2009; 191:1359–1360. 10.1128/JB.01682-08 19074396 PMC2632009

[20] Silva AJ, Benitez JA. Th1-type immune response to a *Coccidioides immitis* antigen delivered by an attenuated strain of the non-invasive enteropathogen *Vibrio cholerae*. FEMS Immunol Med Microbiol. 2005; 43:393–398. 10.1016/j.femsim.2004.10.001 15708313

[21] Ghasemi A, Jeddi-Tehrani M, Mautner J, Salari MH, Zarnani AH. Simultaneous immunization of mice with Omp31 and TF provides protection against *Brucella melitensis* infection. Vaccine. 2015; 33:5532–5538. 10.1016/j.vaccine.2015.09.013 26384448

[22] Zhou H, Yang B, Xu F, Chen X, Wang J, Blackall PJ, et al Identification of putative virulence-associated genes of *Haemophilus parasuis* through suppression subtractive hybridization. Vet Microbiol. 2010; 144:377–383. 10.1016/j.vetmic.2010.01.023 20171024

[23] Tadjine M, Mittal KR, Bourdon S, Gottschalk M. Production and characterization of murine monoclonal antibodies against *Haemophilus parasuis* and study of their protective role in mice. Microbiology. 2004; 150:3935–3945. 10.1099/mic.0.27443-0 15583147

[24] Zheng N, Chai Z, Fu F, Jiang F, Wang X, Zhang X, et al Identification of a novel *Haemophilus parasuis*-specific B cell epitope using monoclonal antibody against the OppA protein. PLoS One. 2014; 9:e84516 10.1371/journal.pone.0084516 24416241 PMC3887010

[25] Takahashi K, Nagai S, Yagihashi T, Ikehata T, Nakano Y, Senna K, et al A cross-protection experiment in pigs vaccinated with *Haemophilus parasuis* serovars 2 and 5 bacterins, and evaluation of a bivalent vaccine under laboratory and field conditions. J Vet Med Sci. 2001; 63:487–491. 11411491 10.1292/jvms.63.487

[26] Doyle CR, Pan JA, Mena P, Zong WX, Thanassi DG. TolC-dependent modulation of host cell death by the *Francisella tularensis* live vaccine strain. Infect Immun. 2014; 82:2068–2078. 10.1128/IAI.00044-14 24614652 PMC3993420

[27] Thomas S, Holland IB, Schmitt L. The Type 1 secretion pathway—the hemolysin system and beyond. Biochim Biophys Acta. 2014; 1843:1629–1641. 10.1016/j.bbamcr.2013.09.017 24129268

[28] Hou B, Meng XR, Zhang LY, Tan C, Jin H, Zhou R, et al TolC promotes ExPEC biofilm formation and curli production in response to medium osmolarity. Biomed Res Int. 2014; 2014:574274 10.1155/2014/574274 25243151 PMC4163439

[29] Yang TC, Ma XC, Liu F, Lin LR, Liu LL, Liu GL, et al Screening of the *Salmonella paratyphi A* CMCC 50973 strain outer membrane proteins for the identification of potential vaccine targets. Mol Med Rep. 2012; 5(1):78–83. 10.3892/mmr.2011.587 21922141

[30] Kovacs-Simon A, Titball RW, Michell SL. Lipoproteins of bacterial pathogens. Infect Immun. 2011; 79:548–561. 10.1128/IAI.00682-10 20974828 PMC3028857

[31] Okay S, Özcengiz E, Gürsel I, Özcengiz G. Immunogenicity and protective efficacy of the recombinant *Pasteurella* lipoprotein E and outer membrane protein H from *Pasteurella multocida* A:3 in mice. Res Vet Sci. 2012; 93:1261–1265. 10.1016/j.rvsc.2012.05.011 22727197

[32] Martins VT, Chávez-Fumagalli MA, Lage DP, Duarte MC, Garde E, Costa LE, et al Antigenicity, immunogenicity and protective efficacy of three proteins expressed in the promastigote and amastigote stages of Leishmania infantum against visceral Leishmaniasis. PLoS One. 2015; 10:e0137683 10.1371/journal.pone.0137683 26367128 PMC4569552

[33] Golshani M, Rafati S, Dashti A, Gholami E, Siadat SD, Oloomi M, et al Vaccination with recombinant L7/L12-truncated Omp31 protein induces protection against *Brucella* infection in BALB/c mice. Mol Immunol. 2015; 65:287–292. 10.1016/j.molimm.2015.01.009 25723468

[34] Williamson ED, Packer PJ, Waters EL, Simpson AJ, Dyer D, Hartings J, et al Recombinant (F1 + V) vaccine protects cynomolgus macaques against pneumonic plague. Vaccine. 2011; 29:4771–4777. 10.1016/j.vaccine.2011.04.084 21570437

[35] Vance DJ, Rong Y, Brey RN 3rd, Mantis NJ. Combination of two candidate subunit vaccine antigens elicits protective immunity to ricin and anthrax toxin in mice. Vaccine. 2015; 33:417–421. 10.1016/j.vaccine.2014.11.036 25475957 PMC4274239

[36] Tanabe M, Atkins HS, Harland DN, Elvin SJ, Stagg AJ, Mirza O, et al The ABC transporter protein OppA provides protection against experimental *Yersinia pestis* infection. Infect Immun. 2006; 74:3687–3691. 10.1128/IAI.01837-05 16714605 PMC1479284

[37] Yang M, Johnson A, Murphy TF. Characterization and evaluation of the *Moraxella catarrhalis* oligopeptide permease A as a mucosal vaccine antigen. Infect Immun. 2011; 79:846–857. 10.1128/IAI.00314-10 21134967 PMC3028828

[38] Chen S, Chu Y, Zhao P, He Y, Jian Y, Liu Y, et al Development of a recombinant OppA-based indirect hemagglutination test for the detection of antibodies against *Haemophilus parasuis*. Acta Trop. 2015; 148:8–12. 10.1016/j.actatropica.2015.04.009 25910625

[39] Morton DJ, Seale TW, Madore LL, VanWagoner TM, Whitby PW, Stull TL. The haem-haemopexin utilization gene cluster (hxuCBA) as a virulence factor of *Haemophilus influenzae*. Microbiology. 2007; 153(Pt 1):215–224. 10.1099/mic.0.2006/000190-0 17185550

